# The clinical validity of atlantoaxial joint inclination angle and reduction index for atlantoaxial dislocation

**DOI:** 10.3389/fsurg.2022.1028721

**Published:** 2023-01-06

**Authors:** Yang Qu, Yukun Du, Yonghua Zhao, Jianyi Li, Hao Luo, Jiaxiang Zhou, Yongming Xi

**Affiliations:** Department of Spinal Surgery, The Affiliated Hospital of Qingdao University, Qingdao, China

**Keywords:** reduction index, atlantoaxial joint angle, atlas-dens interval, operation approach, atlantoaxial dislocation

## Abstract

**Objective:**

Atlantoaxial dislocation patients with neurological defects require surgery. Sometimes, release surgery is necessary for irreducible atlantoaxial dislocation to further achieve reduction. Whether release surgery is essential relies on the surgeon's experience and lacks objective reference criteria. To evaluate the value of atlantoaxial joint inclination angle (AAJI) in sagittal and coronal planes and reduction index (RI) in the surgical approach selection for atlantoaxial dislocation.

**Methods:**

Retrospectively analyzed 87 cases (42 males and 45 females, 9–89 years) of atlantoaxial dislocation from January 2011 to November 2020. In addition, 40 individuals without atlantoaxial dislocation were selected as the control group. Imaging parameters were compared between the two groups. According to surgical methods, the experiment group was divided into two groups including Group A(release surgery group) and Group B (conventional operation group). The parameters were measured based on CT and x-ray. The relevant imaging parameters and clinical scores, including the AAJI in sagittal and coronal planes, the atlas-dens interval (ADI) before and after traction, the RI, and JOA scores were measured and analyzed.

**Results:**

The sagittal and coronal atlantoaxial joint inclination angles(SAAJI and CAAJI) in the control group were 7.91 ± 0.42(L), 7.99 ± 0.39°(R), 12.92 ± 0.41°(L), 12.97 ± 0.37°(R), in A were 28.94 ± 1.46°(L), 28.57 ± 1.55°(R), 27.41 ± 1.29°(L), 27.84 ± 1.55°(R), and in B were 16.16 ± 0.95°(L), 16.80 ± 1.00°(R), 24.60 ± 0.84°(L), 24.92 ± 0.93°(R) respectively. Statistical analysis showed that there was a statistical difference in the SAAJI between the control group and the experiment group (*P* < 0.01), as well as between groups A and B (*P* < 0.01). The RI in groups A and B was 27.78 ± 1.46% and 48.60 ± 1.22% respectively, and there was also a significant difference between the two groups (*P* < 0.01). There was negative correlation between SAAJI and RI.

**Conclusions:**

The SAAJI and RI can be used as objective imaging indexes to evaluate the reducibility of atlantoaxial dislocation. And these parameters could further guide the selection of surgery methods. When the RI is smaller than 48.60% and SAAJI is bigger than 28.94°, anterior release may be required.

## Introduction

Atlantoaxial dislocation(AAD) refers to the stability loss of the atlantoaxial joint, resulting in the abnormal atlantoaxial structure (1). AAD can be caused by various reasons, including inflammation, tumor, trauma, odontoid fracture, congenital developmental deformity, and rupture of the transverse ligament. It can lead to neurological symptoms and paralysis. AAD could be divided into three types in clinical: traction reduction type, operation reduction type and irreducible type. Irreducible atlantoaxial dislocation (IAAD) is one of the types of AAD, which is difficult to reduction due to various factors, including fibrous scars, osteophyte formation, and even bony barrier. Traditionally, the diagnosis of IAAD can be made by observing the dynamic position x-ray to judge the difficulty of reduction under the dynamic position. But different opinions have been raised by some surgeons. Wang C et al. indicated that IAAD was considered if large weight (1/6 bodyweight) cranial traction after anesthesia was not able to achieve the reduction while the preoperative CT showed the absence of C1–2 lateral mass joint fusion (2). Salunke et al. believed that traction should start at 7% of body weight and gradually increase traction weight to 20% of maximum bodyweight within 48–72 h (3). It was generally believed that preoperative cranial traction was necessary for the diagnosis of IAAD.

In the treatment of AAD, decompression and maintaining regional stability of the cervical spine are the basic requirements of the operation. For some IAAD patients, posterior reduction and fixation cannot achieve complete reduction, and release sugery is necessary. The routine surgical procedures for atlantoaxial dislocation are as follows: (1) Anterior release and posterior fixation; (2) posterior reduction and fusion; (3) posterior release and fixation (4–6). At present, the management of release surgery is mainly based on the surgeon's clinical experience, lacking objective imaging reference standards (7). But there was controversial about conduction of release surgery for IAAD, which increases the blindness of treatment choice of AAD (8, 9). It has been found in clinical practice that many factors affect the reduction of AAD. Salunke et al. proposed that the angle of the atlantoaxial joint surface is of great significance in evaluating the difficulty of AAD in the sagittal plane. Based on our experience, we found that there is the correlation between atlantoaxial joint inclination angle (AAJI) and AAD. It is considered that the greater the angle and amplitude of forward tilt of atlas, the higher the relative difficulty of reduction. Therefore, it is crucial to establish an accurate and relatively objective imaging criterion for evaluating the difficulty of AAD reduction (10). Based on this, our previous study proposed a new concept named sagittal atlantoaxial joint inclination angle (SAAJI) and reduction index (RI), which can be used as an objective imaging criterion to guide the selection of surgical methods, but the study sample size was small. Aiming to further evaluate the significance of AAJI in evaluating the difficulty of atlantoaxial reduction, the CT and x-ray were conducted retrospectively to analyze the clinical value of AAJI and RI for AAD, and to provide surgeons with objective standards helping the selection of surgical procedures.

## Materials and methods

### Patients

With the approval of the ethics committee of our hospital, the patients who signed the informed consent form were included in this study. A total of 87 patients with AAD and 40 patients with normal cervical vertebra structure, from January 2011 to November 2020, were enrolled in this study. The inclusion criteria are as follows: (1) The patients with AAD received traction before the operation; (2) The patients had no oral or periodontal-related diseases; (3) Imaging examination is complete; (4) The patients were followed up for at least 12 months. The exclusion criteria are as follows: (1) The patients did not receive traction before operation; (2) Patients with coagulation system diseases or other severe comorbidities; (3) Lack of important examination; (4) The follow-up time was less than 12 months; (5) Patients with severe osteoporosis (T ≤ −2.5). The surgery selection criteria are as follows: when the intraoperative skull traction cannot get satisfied reduction, the release and reduction operation will be performed, otherwise conventional reduction operation will be performed. The 40 patients with normal cervical vertebra structure were regarded as the control group. Based on the surgical procedure, the 87 patients were further divided into two groups: Group A (release surgery group) and Group B (conventional operation group).

### Clinical evaluation

Select reasonable statistical methods to sort out and compare the basic information of patients in groups A and B, then clarify whether there are statistical differences in the proportion of men and women and the average age of patients. All patients used the latest cervical Japanese Orthopaedic Association (JOA) scores to evaluate the degree of cervical spinal symptoms and calculated the improvement rate of cervical spinal function after treatment. The formula of JOA improvement rate (IR) was (postoperative total score-preoperative total score)/(17-preoperative total score) × 100%.

### Radiological evaluation

**Figure 1:**
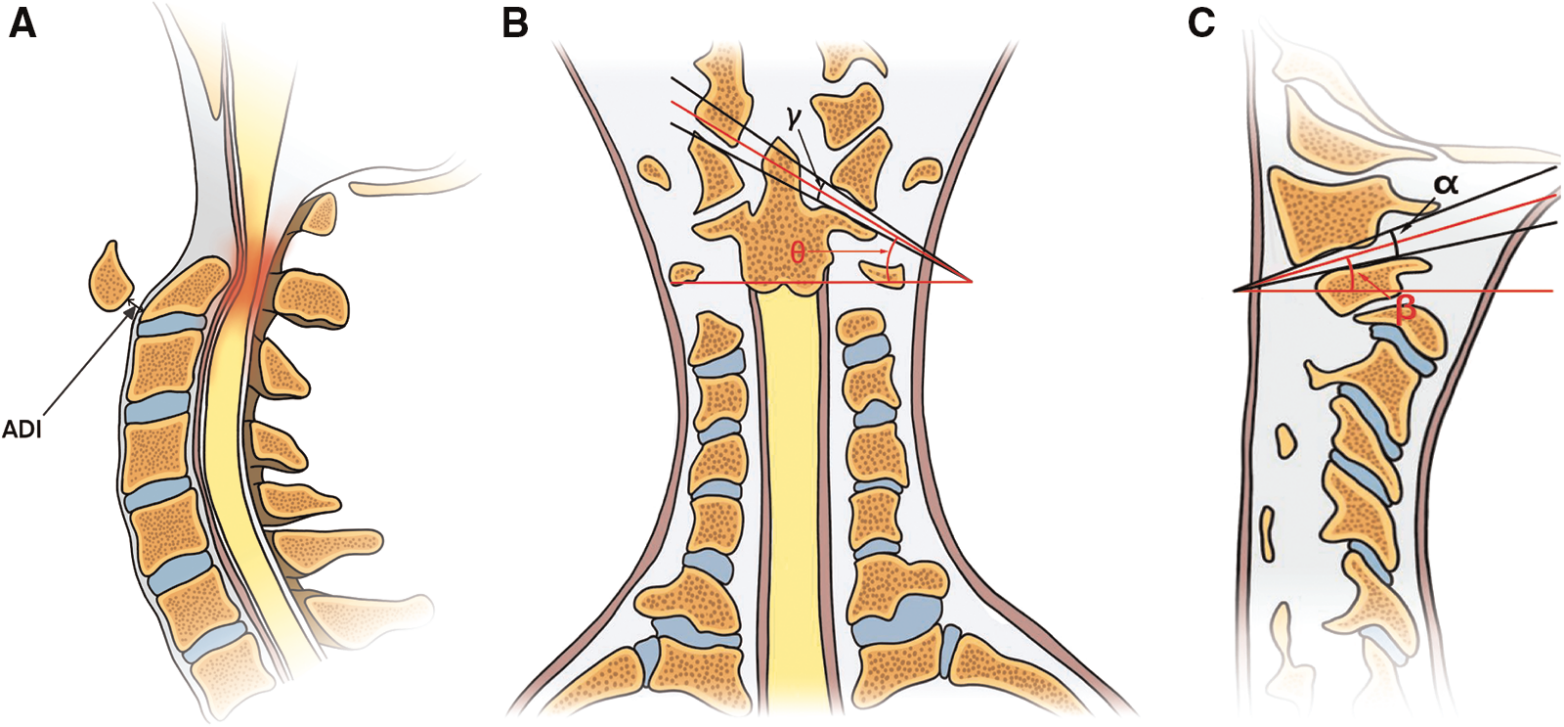
(**A**) The measurement of atlas-dens interval, the red section refers to the squeezed spine; (**B**) Angle *γ* is formed by the tangents lines of the articular surface, the angle *θ* refers to the coronal atlantoaxial joint inclination angle; (**C**) angle *α* is similar to angle *γ*, the angle *β* refers to the sagittal atlantoaxial joint inclination angle.

**Figure 2:**
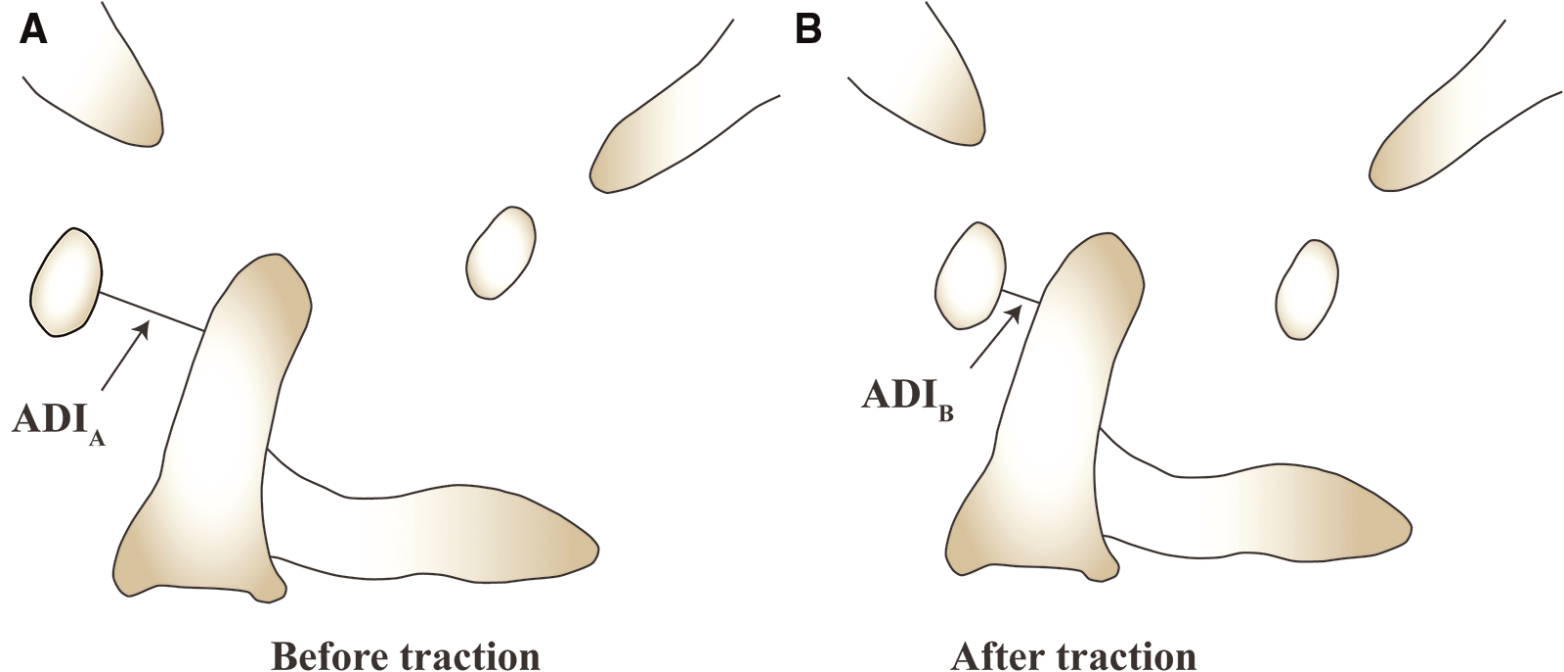
(**A**) The ADI measure before the traction(marked with ADI_A_); (**B**) The ADI measured after traction(marked with ADI_B_). RI can be calculated by this formulate: RI = (ADI_A_–ADI_B_)/ADI_A._

Atlas-dens interval (ADI) and reduction index (RI): The vertical distance from the posterior edge of the anterior arch of the atlas to the tangent line of the odontoid was measured in the anterior and lateral cervical x-ray examination, that is the ADI (Figure 1A). Then, according to the change of the ADI before and after traction, the RI can be calculated, and the calculation formula is (pre-traction ADI—post-traction ADI)/pre-traction ADI*100% (Figure 2).

Coronal Atlantoaxial joint inclination angle (CAAJI): similar to the sagittal plane, using the bone window of CT scan, the tangents of inferior articular surface of lateral mass of atlas and superior articular surface of axis were made in coronal plane, and the included angle of the two tangents was angle *γ*. The angle which was formed by the bisector of angle *γ* and the horizontal line was the angle *θ* (Figure 1B).

Sagittal Atlantoaxial joint inclination angle (SAAJI): using the bone window of CT scan of cervical vertebra, the tangents of inferior articular surface of lateral mass of atlas and superior articular surface of axis were made in sagittal plane, and the included angle of the two tangents was angle *α*. The angle was formed by the bisector of angle *α* and the horizontal line was the angle *β* (Figure 1C).

### Statistical analysis

Using SPSS 26.0 version software (SPSS Inc, Chicago, Illinois, USA.) for data analysis. The normality of the data was first tested by the Shapiro-Wilk test. All data are presented as the means and standard deviations in both chart and words. The statistical significance threshold was *P* < 0.05. In addition, Spearman correlation analysis was also conducted to get the correlation and correlation degree between parameters.

## Result

### Patient cohort

A total of 87 patients with AAD were included in the experiment group. And we statistically analyzed the information between Group A and B. The average age in group A was 57.8 ± 2.6 years (range 28–78 years), with 57.1% females and 42.9% males. While in the group B, the average age was 54.5 ± 2.1 years (range 9–89 years), with 49.2% females and 50.8% males. There were 28 patients in group A, 59 patients in group B. As we can see through the analysis, there are no statistical difference between Group A and B in both age and sex. In addition, the results of control group showed that the average age is 59.3 ± 1.9 years (range 35–76), with 45.0% females and 55.0% males (Table 1).

**Table 1:** The general patients information.

Group	Case	Age	Sex (M:F)	IR
A	28	57.75 ± 2.60	12:16	81.02 ± 2.23%
B	59	54.54 ± 2.13	30:29	79.52 ± 1.82%
*P*-Value		0.372	0.486	0.624

IR, JOA improvement rate.

### Atlantoaxial joint inclination angle

In the control group, the AAJI in sagittal plane was 7.95 ± 0.28°, in coronal plane was 12.94 ± 0.28°. In group A, the average AAJI in coronal plane was 27.41 ± 1.29°(L) and 27.84 ± 1.55°(R), in sagittal plane is 28.94 ± 1.46°(L) and 28.57 ± 1.55°(R). In group B, the average AAJI in coronal plane was 24.60 ± 0.84°(L) and 24.92 ± 0.93°(R), in sagittal plane was 16.16 ± 0.95°(L) and 16.80 ± 1.00°(R). Statistical analysis showed that there was significant difference in AAJI between control group and experiment group (*P* < 0.01). Meanwhile, there was also significant difference in AAJI between group A and B (*P* < 0.01) (Table 2).

**Table 2:** Comparison of key parameters.

Variable/parameters	Group A	Group B	*P* Value
SAAJI (L)	28.94 ± 1.46°	16.16 ± 0.95°	<0.01
SAAJI (R)	28.57 ± 1.55°	16.80 ± 1.00°	<0.01
CAAJI (L)	27.41 ± 1.29°	24.60 ± 0.84°	<0.01
CAAJI (R)	27.84 ± 1.55°	24.92 ± 0.93°	<0.01
JOA (Pre-O)	8.50 ± 0.35	8.53 ± 0.21	0.948
JOA (Post-O)	15.21 ± 0.25	15.15 ± 0.18	0.843
ADI (Pre-T)	6.49 ± 0.22	7.06 ± 0.24	0.142
ADI (Post-T)	4.74 ± 0.22	3.70 ± 0.18	<0.01
RI	27.78 ± 1.46%	48.60 ± 1.22%	<0.01

SAAJI, sagittal atlantoaxial joint inclination angle; CAAJI, coronal atlantoaxial joint inclination angle; JOA (Pre-O), preoperative JOA scores; JOA (Post-O), postoperative JOA scores; ADI(Pre-T), ADI before traction; ADI(Post-T), ADI after traction; RI, reduction index; L, left; R, right.

### ADI and reduction index

The average ADI before and after traction in group A is 6.49 ± 0.22 and 4.74 ± 0.22 respectively, reduction index is 27.78 ± 1.46%; then the average ADI before and after traction in group B is 7.06 ± 0.24 and 3.70 ± 0.18 respectively, reduction index is 48.60 ± 1.22%. Statistical analysis showed that there was no statistical difference in ADI before traction between group A and group B, but the reduction index in group A and group B were significantly different (*P* < 0.01) (Table 2).

### JOA score

Among the included patients with AAD, we made a comparative analysis of JOA scores between group A and group B. In group A, the preoperative JOA scores were 8.50 ± 0.35, the postoperative JOA scores was 15.21 ± 0.25, the average JOA score improvement rate was 81.02 ± 2.23%. In group B, the average preoperative JOA scores were 8.53 ± 0.21, the average postoperative JOA score was 15.15 ± 0.18, and the average improvement rate was 79.52 ± 1.82% (Table 2). This showed that the spinal cord function was improved, the symptoms were relieved in both groups after the operation, and there was no statistical difference in JOA improvement rates between two groups (Table 1).

### The correlation between ri and AAJI

The results of the correlation analysis showed that SAAJI of groups A and B were negatively correlated with the RI (*P* < 0.01), the correlation coefficient index is −0.731, indicating that the smaller the atlantoaxial inclination angle was, the larger the reduction index was. In contrast, CAAJI of both group A and group B were not significantly correlated with the RI. RI represents the difficulty of reduction. This showed that when the atlantoaxial inclination angle in sagittal is bigger, the difficulty of reduction is bigger.

### Case presentation

**Figure 3:**
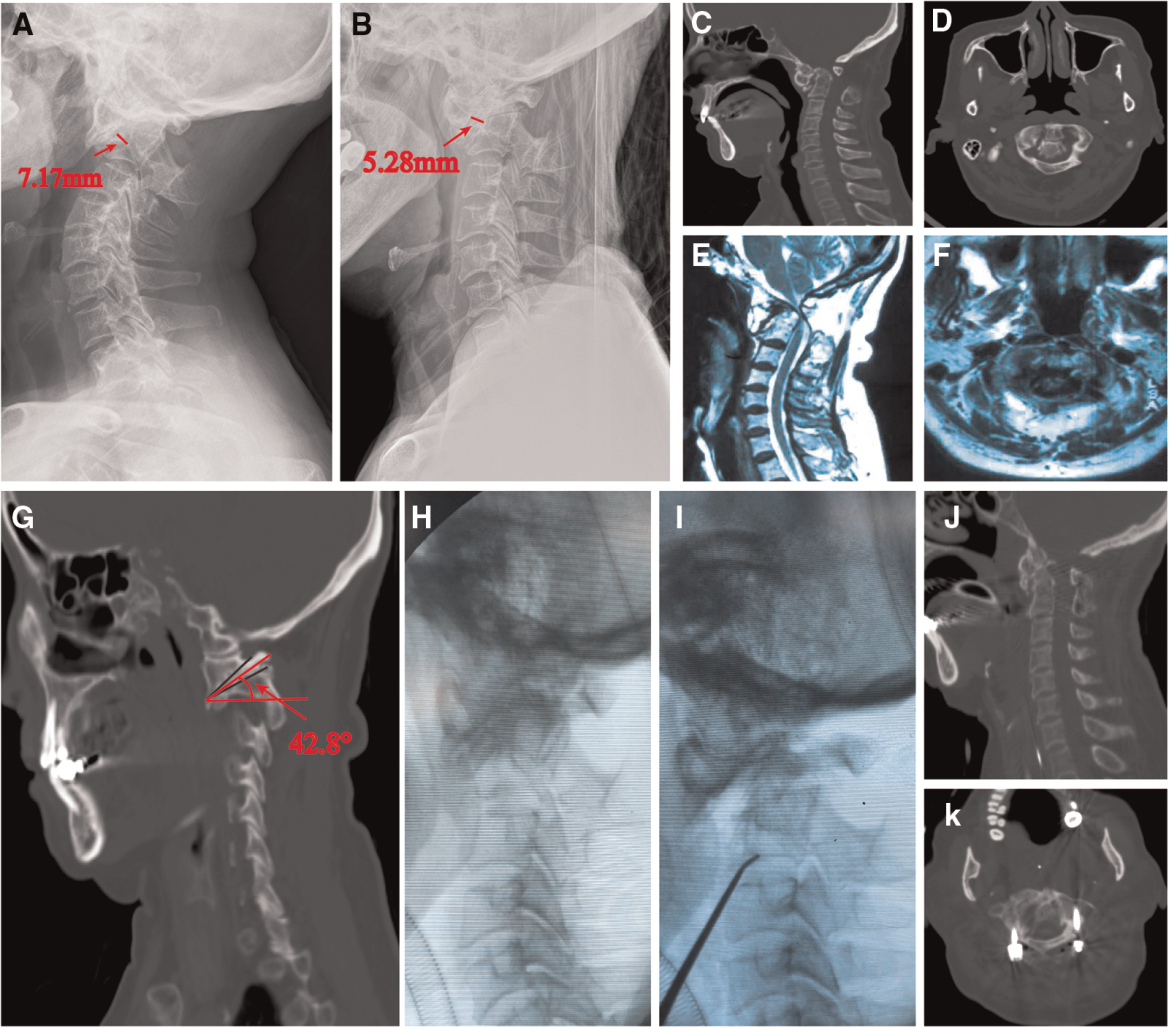
(**A,B**) A 71 years old female with AAD, the x-ray showed the results of traction: ADI before and after traction is 7.17 mm and 5.28 mm, RI is 26.36%. (**C,D**) Computed tomography (CT) was taken to show the condition of dislocation and fusion. (**E,F**) Magnetic resonance imaging (MRI) showed the compression of cervical spine. (**G**) We measured the SAAJI in the sagittal CT, and the result is 42.8°. (**H–K**) Anterior release and posterior operation was conducted for her, which get a satisfied reduction.

**Figure 4:**
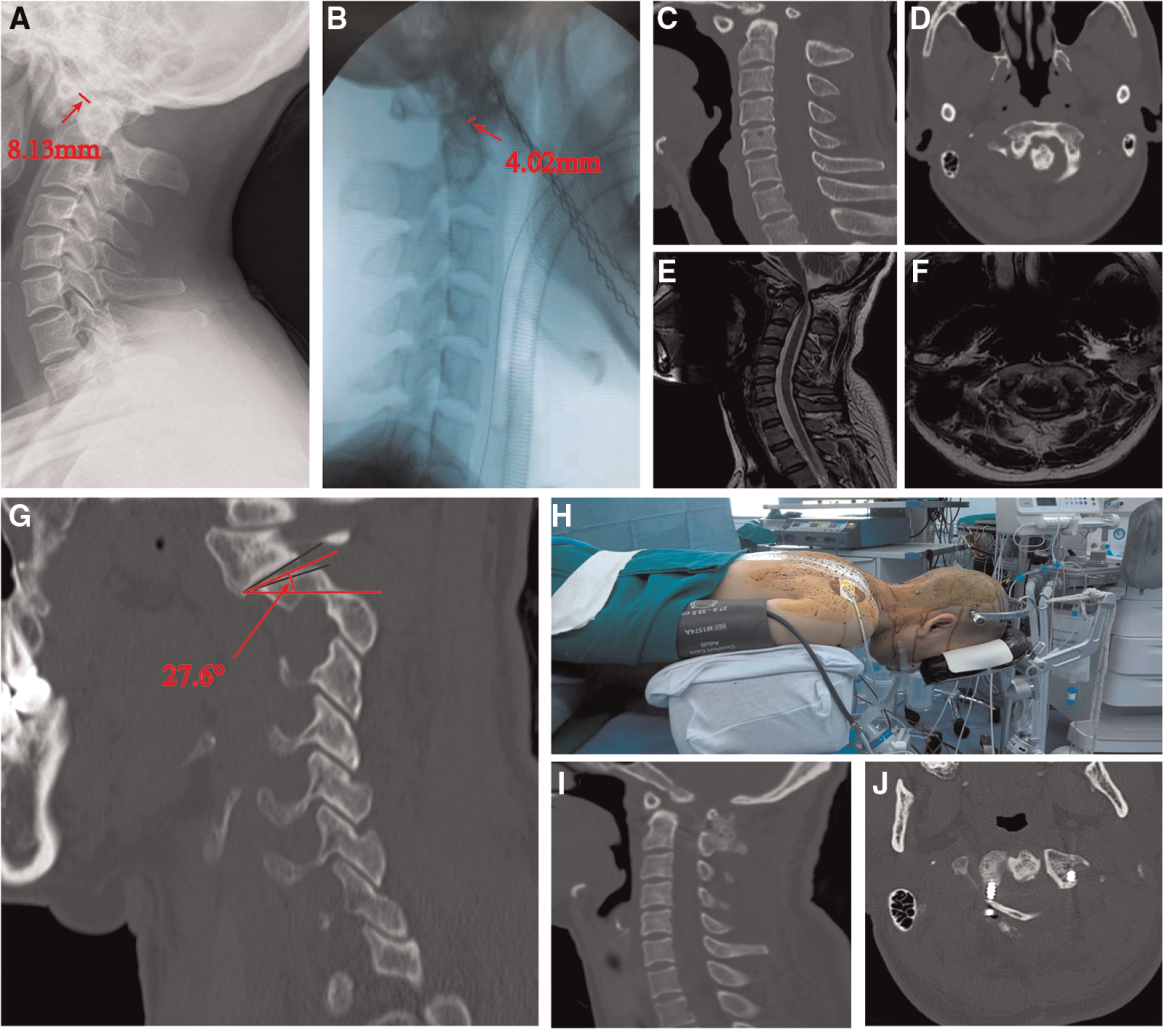
(**A,B**) A 42 years old female with AAD, traction was also conducted for her to evaluate the difficulty of reduction, which get the ADI before and after traction is 8.13 mm and 4.12 mm, RI is 50.55%. (**C–F**) CT and MRI showed that the fusion is not too much and the cervical spine is compressed severely. (**H–J**) The SAAJI was also measured, the result is 27.6° (**G**). We conducted a posterior operation with traction and get satisfied reduction.

We select two typical AAD patients who were treated with the guide of our findings. Patient 1: It's a 71 years old female with AAD who suffered from progressive numbness and weakness in the limbs for more than 2 years. According to our measurement, the ADI before and after traction is 7.17 mm and 5.28 mm, SAAJI is 42.8°, RI is 26.36%, and JOA score is 6. With the guide of the standard we raised, we conducted an anterior release and posterior fixation operation for her, which resulted in a satisfied reduction and relief in symptoms (Figure 3). After the surgery, the JOA score is 12. Patient 2: It's a 45 years old female with AAD whose ADI before and after traction is 8.13 mm and 4.02 mm, SAAJI is 27.6°, RI is 50.55% and JOA score is 7. We conducted a posterior operation for her, which also resulted in satisfied reduction and relief in symptoms (Figure 4). After this surgery, the JOA score is 14.

## Discussion

Atlantoaxial dislocation is a rare and potentially fatal anatomical disorder of the occipitocervical region leading to permanent neurological defects or sagittal deformities without timely treatment (1). For AAD, the traditional operation is one-stage posterior reduction and internal fixation (11). But for IAAD, release surgery may be necessary (11, 12). At present, anterior release includes transoral approach, transnasal approach and submandibular approach (2, 13–15). Yin QS et al. proposed the TARP plate system to perform decompression, reduction, internal fixation through the transoral approach (16–20). Wang et al. promoted transoral atlantoaxial release combined with posterior fixation to treat irreducible atlantoaxial dislocation (2). However, the transoral approach will increase the risk of infection during the perioperative period. Some patients with oral diseases are unable to complete transoral release surgery. Combined anterior and posterior surgery also has some limitations. It will cause long operation time, high risk, complicated situation, and higher cost (21). Moreover, posterior release surgery can also achieve good results in some cases with the development of surgical techniques. By improving Goel technique, Chen Z et al. performed one-stage posterior joint release on patients with atlantoaxial dislocation with bony fusion and reduced the dislocated atlantoaxial vertebra (22). Although a variety of surgical methods, the choice of surgical methods is mainly based on the surgeon's clinical experience (23). Meanwhile, anterior release is also controversial (15). As far as we know, there was few research on the objective criteria for the selection of surgical methods for atlantoaxial dislocation.

The atlantoaxial joint is responsible for a large part of the movement of the neck, and these movements usually occur in different planes. Salunke et al. objectively evaluated the dislocation of the first and second cervical vertebrae in multiple planes and discussed the possible mechanisms and methods for the reduction of various types of dislocation (24). Chandra et al. firstly indicated the correlation between the position of the atlantoaxial joint and the severity of the AAD. They also described the new indexes named “sagittal joint inclination” to describe the position and shape of the atlantoaxial joint (25). Baoge Liu et al. also studied the changes of related parameters of the atlantoaxial joint and atlantooccipital joint before and after anterior cervical surgery and discussed the role of related parameters of cervical spine in treatment and evaluation of curative effect (26). For spinal surgeons, it is important to understand the position and shape of atlantoaxial joints in normal people for better knowing the correction process of AAD. Therefore, it is worthy to dictate criteria for the selection of reasonable surgery methods according to the parameters.

With regard to the AAJI involving in this study, Salunke et al. proposed that an angle between the tangent lines of the atlantoaxial joint surface is of significance for the evaluation of the severity of atlantoaxial dislocation. In that study, 24 patients were included and measured. He considered that the larger angle predicted the higher severity of atlantoaxial dislocation (3). But the study lacks the further research of correlation between the angle and the difficulty of reduction. Chandra et al. have confirmed that the atlantoaxial inclination angle was related to the severity and difficulty of reduction, which was of great significance in distinguishing and judging irreducible atlantoaxial dislocation and reducible atlantoaxial dislocation. This study also confirmed that the atlantoaxial inclination angle on the coronal plane was related to the severity of basilar invagination (25). The above studies only researched the relationship between imaging parameters and the severity of the atlantoaxial dislocation without guiding the selection of surgical methods. Furthermore, the measurement of angles was merely based on the plane conversion. Another index involved in this study is the ADI, which refers to the vertical distance from the leading edge of the posterior arch of the atlas to the tangent line of the leading edge of the odontoid, which is a pivotal measurement parameter for the diagnosis of AAD. Our former study put forward the concept of RI by studying the AAD, which is calculated by the ADI before and after traction. It is used to express the degree of atlantoaxial reduction in this plane after traction (27).

In this study, based on the influence of multi-dimensional stability of atlantoaxial joint on reduction, the SAAJI was measured, and CAAJI was added to analyze the effect of coronal angle on the difficulty of reduction. The RI was used to analyze the correlation between the reduction index and the inclination angle of coronal plane and sagittal plane. Through the analysis of imaging and clinical knowledge, we found that patients in group A had larger inclination angle of sagittal plane (28.94 ± 1.46°) and smaller reduction index (27.78 ± 1.46%) than patients in group B, which were respectively 16.80 ± 1.00° and 48.60 ± 1.22%. In other words, higher inclination angle may indicate the higher difficulty of reduction. For these patients, anterior release is necessary to ensure the reduction and the decompression of spinal cord. Compared with the previous studies, this study not only added the CAAJI as a new evaluation index but also included more patients, which further improved the accuracy and reliability of this study.

What's more, multiple regression analysis showed that the RI was negatively correlated with the SAAJI, but there was no relation with CAAJI. Through these parameters, we can judge the difficulty of reduction of AAD and guide the determination of surgery. For example, if the SAAJI is more than the average in group A (28.94 ± 1.46°), RI is less than the average level of group B (48.60 ± 1.22%), release surgery may be required. Otherwise, the single-stage posterior reduction surgery is feasible. This study improved the evaluation level of SAAJI and RI for judging the difficulty of atlantoaxial dislocation reduction, and further proved that the coronal atlantoaxial inclination angle is invalid. Furthermore, we found that in group A, the SAAJI of some patients cannot meet the standard of release surgery, but the RI is small, and obvious bone fusion can be seen in CT images. For this kind of patients, the anterior release also can be conducted based on the actual clinical situation. Through the study, a more clinically valuable objective standard for the selection of surgical methods for AAD can assist doctors in deciding the management of anterior release. It also has advantages in avoiding secondary operation, reducing the cost and risk of the operation.

In this study, there are still some limitations. It is still necessary to expand the samples quantity and improve the reliability of the SAAJI and RI. According to the conclusions of this study, prospective experiments need to be carried out in the future to improve the credibility. In addition, the selection of surgery method may also have influence on the division of group. The atlantoaxial joint is a multi-dimensional structural complex, so we still need to study more parameters in order to improve the ability of the model to simulate the real situation.

## Conclusion

In this study, there is negative correlation between SAAJI and RI. The SAAJI indicated the severity of AAD, the RI indicated the difficulty of reduction. When the RI is smaller than 48.60% and SAAJI is bigger than 28.94°, anterior release may be required. The atlantoaxial joint inclination angle and reduction index can be used as objective criteria to guide the selection of surgical methods for atlantoaxial dislocation.

## Data Availability

The raw data supporting the conclusions of this article will be made available by the authors, without undue reservation.
